# Transcriptome Analysis of *Taxillusi chinensis (DC*.*) *Danser Seeds in Response to Water Loss

**DOI:** 10.1371/journal.pone.0169177

**Published:** 2017-01-03

**Authors:** Shugen Wei, Xiaojun Ma, Limei Pan, Jianhua Miao, Jine Fu, Longhua Bai, Zhonglian Zhang, Yanhong Guan, Changming Mo, Hao Huang, Maoshan Chen

**Affiliations:** 1 Institute of Medicinal Plant Development, Chinese Academy of Medical Sciences & Peking Union Medical College, Beijing, China; 2 Guangxi Botanical Garden of Medicinal Plants, Nanning, Guangxi, China; 3 Yunnan Branch Institute of Medicinal Plant Development, Chinese Academy of Medical Science, Jinghong, China; 4 Department of Biochemistry and Genetics, La Trobe Institute for Molecular Science (LIMS), La Trobe University, Melbourne, Victoria, Australia; Kumamoto University, JAPAN

## Abstract

**Background:**

*Taxillus chinensis (DC*.*)* Danser, the official species of parasitic loranthus that grows by parasitizing other plants, is used in various traditional Chinese medicine prescriptions. ABA-dependent and ABA-independent pathways are two major pathways in response to drought stress for plants and some genes have been reported to play a key role during the dehydration including dehydration-responsive protein RD22, late embryogenesis abundant (LEA) proteins, and various transcription factors (TFs) like MYB and WRKY. However, genes responding to dehydration are still unknown in loranthus.

**Methods and Results:**

Initially, loranthus seeds were characterized as recalcitrant seeds. Then, biological replicates of fresh loranthus seeds (CK), and seeds after being dehydrated for 16 hours (Tac-16) and 36 hours (Tac-36) were sequenced by RNA-Seq, generating 386,542,846 high quality reads. A total of 164,546 transcripts corresponding to 114,971 genes were assembled by Trinity and annotated by mapping them to NCBI non-redundant (NR), UniProt, GO, KEGG pathway and COG databases. Transcriptome profiling identified 60,695, 56,027 and 66,389 transcripts (>1 FPKM) in CK, Tac-16 and Tac-36, respectively. Compared to CK, we obtained 2,102 up-regulated and 1,344 down-regulated transcripts in Tac-16 and 1,649 up-regulated and 2,135 down-regulated transcripts in Tac-36 by using edgeR. Among them some have been reported to function in dehydration process, such as RD22, heat shock proteins (HSP) and various TFs (MYB, WRKY and ethylene-responsive transcription factors). Interestingly, transcripts encoding ribosomal proteins peaked in Tac-16. It is indicated that HSPs and ribosomal proteins may function in early response to drought stress. Raw sequencing data can be accessed in NCBI SRA platform under the accession number SRA309567.

**Conclusions:**

This is the first time to profile transcriptome globally in loranthus seeds. Our findings provide insights into the gene regulations of loranthus seeds in response to water loss and expand our current understanding of drought tolerance and germination of seeds.

## Introduction

*Taxillus chinensis (DC*.*)* Danser, which is the official name of parasitic loranthus according to the Pharmacopoeia, is wildly used in various traditional Chinese medicine prescriptions such as the treatment of rheumatism, threatened abortion, hypertension, angina pectoris, stroke, and arrhythmia for many years in China [1–4]. There are a total of 51 species of parasitic loranthus, of which 23 are distributed in Guangxi of China [5]. It is also called “Sangjisheng” in China and grows by parasitizing other plants like Aceraceae, Anacardiaceae, Euphorbiaceae, Fabaceae, Fagaceae, Juglandaceae, Moraceae, Rosaceae, and Rutaceae [6]. In plants, developmental processes such as seed germination, seedling development, leaf development and flowering are always affected by various environmental stresses, such as drought, high salinity, and high or low temperatures [7–9]. However, it is still unknown about the effects of these environmental stresses on loranthaceous.

In plants, drought stress induces various biochemical and physiological responses, such as stomatal closure, repression of cell growth and photosynthesis, and activation of respiration [10]. At cellular and molecular levels, a large number of genes have been reported to respond to drought stress [11–13]. Large scale profiling methods like microarray and next-generation sequencing have been demonstrated to estimate the gene expression changes during dehydration process in several model species, such as *Arabidopsis* [14, 15], rice [16–18], soybean [19, 20] and other plants [21–23]. These studies have shown that the plant defense against drought stress starts with the perception of water loss, which can trigger the activation of abscisic acid (ABA)-dependent and ABA–independent regulatory systems [24]. According to their functions, the gene products induced by drought stress can be divided into two groups [12, 13, 24]. The first group includes proteins directly protecting against the drought stress, such as chaperones, late embryogenesis abundant (LEA) proteins, mRNA-binding proteins, water channel proteins, and lipid-transfer proteins. The second group contains various TFs that probably function in further regulation of signal transduction and gene expression, protein kinases, protein phosphatases, and other signaling molecules such as calmodulin-binding proteins.

RNA-Seq, a next generation sequencing technology, has become a useful tool for genome-wide gene expression analysis [25]. It enables the *de novo* assembly and gene expression analyses for those species, of which the genome sequences are not available currently [26]. To study drought stress induced genes, RNA-Seq has been used to assemble the transcriptome and profiled gene expression in *Glycine max* [20], *Brassica rapa L*. *ssp*. *Pekinensis* [21], *Bryum argenteum* [27], *Brassica napus* [28], and *Gossypium arboretum* [29]. In Jindou21 (drought-tolerant soybean genotype), 518 and 614 genes including genes ethylene-responsive factors, MYB TFs, and zinc finger proteins are differentially expressed under water deficit condition in leaves and roots, respectively [20]. Comparative transcriptome analysis of *Brassica napus* has shown that a total of 6,018 and 5,377 genes including AREB/ABF, NAC, WRKY and MYB/MYC TFs are induced in response to drought stress [28] in root and leaf, respectively. However, genes induced by drought stress in loranthus are still unknown.

Due to the importance of loranthus in medical use, it is necessary to identify drought responsive genes in loranthus seeds. According to the desiccation tolerance ability, seeds are mainly divided into two types: orthodox and recalcitrant. Recalcitrant seeds lack the mechanisms of metabolic “switch-off” and intracellular dedifferentiation, which contribute significantly to their desiccation sensitivity [30]. On molecular level, the abundance of LEA protein regulated by ABI3 (B3 domain-containing transcription factor) has been verified to link to the desiccation tolerance in recalcitrant and orthodox legume seeds [31]. In this study, we profiled the transcriptome of loranthus seeds during the dehydration using the Illumina HiSeq 2000 system. Differential gene expression analysis and annotation for these transcripts revealed that some gene products have been reported to be involved in drought tolerance, such as various transcription factors, dehydration-responsive protein RD22, ABI3, heat shock proteins and zinc finger proteins. It is interesting that transcripts encoding ribosomal proteins peaked in at loranthus seeds after 16 hours of dehydration. Down-regulation of auxin related proteins, RNA binding protiens, and dehydration-responsive element-binding proteins may be signals of lower germination rates and cell death. This is the first time to analyze transcriptome in loranthus species. Our findings will contribute to understand the drought tolerance mechanism in loranthus and contribute to the research field of loranthus in breeding programs.

## Material and Methods

### Ethics statement

No specific permits were required for the described field studies. The location is not privately-owned or protected in any way, and the field studies did not involve endangered or protected species.

### Seed collection

The seeds of *Taxillus chinensis (DC*.*)* Danser were collected from 10 trees of *Dracontomelon duperreanum* Pierre in Guangxi Province of China in December of 2014. They were confirmed by senior botanists at Institute of Medicinal Plant Development, Chinese Academy of Medical Sciences.

### Seed water loss and water content assay

The dehydration and water content assay of mature loranthus seeds were performed according to the manufacturer’s protocol. In brief, the aluminum case was dried to constant weight, then 100 clean and fresh seeds were weighed (W_1_) within the aluminum case and weighed again after being dried at 100±2°C until constant weight (W_2_). We replicated four times to obtain the average moisture content (W_0_, shown in %) in fresh seeds using the formula below.

$$W_{0}=\frac{W_{1}-W_{2}}{W_{1}}*100\%$$ (1)

Whereas W1 means the average weight of fresh seeds and W2 means the average of dehydrated seeds. Next, another 200 clean and fresh seeds were divided into four groups (50 seeds in each group), incubated in sealed desiccants with silica gel after being weighted and weighted every 4 hours. So the moisture content in seeds after dehydration can be calculated by using this formula:

$$M(\%)=W_{0}-\frac{W_{1}^{\prime }-W_{2}^{\prime }}{W_{1}^{\prime }}*100\%$$ (2)

Here, $W_{1}^{\prime }$ and $W_{2}^{\prime }$ stand for the average weight of fresh seeds before and after dehydration, respectively.

### Determination of seed viability by staining

The viability of loranthus seeds was assessed by immersing the seeds in a solution of 1% (w/v) 2,3,5-Triphenyl Tetrazolium Chloride (TTC, Sigma) according to the protocols [32, 33]. Briefly, using a sterile scalpel 25 seeds were cut for small incisions allowing the TTC to enter. After an eight-hour incubation in 1% TTC solution at 25°C, seeds were washed several times by sterile water. If viable, a redox reaction would change the embryo color from white to reddish brown during cellular respiration [34]. This experiment was replicated four times.

### Germination experiment

Germination experiment was conducted exactly as described previously [35]. Briefly, at each dehydration time point 25 seeds were placed on wetted double layers of Fisher No. 1 filter papers in a dish and incubated at 25°C under 16 h photoperiod for two weeks, before germination rates were determined. Germination experiment was replicated four times for each time point.

### RNA extraction

We selected fresh seeds (CK) and seeds after dehydration for 16 hours (Tac-16) and 36 hours (Tac-36) for deep sequencing. Total RNA was isolated from the seeds by using TRIzol^®^ reagent (Invitrogen) according to the manufacturer’s protocol [36]. Briefly, 10 seeds (~ 3–4 g) was mixed with 1 ml of TRIzol^®^ reagent, homogenized by power homogenizer and centrifuged at 12,000 ×g for 10 min at 4°C. Then, the fatty layer was discarded, cleared supernatant was transferred into a new tube. Next, 0.2 ml of chloroform was added into the tube, following by shaking the tube for 15 secs, centrifugation at 12,000 ×g for 15 min at 4°C and moving the aqueous phase into another new tube for RNA precipitation. We added 10 μg of RNase-free glycogen and 0.5 ml of 100% isopropanol into the aqueous phase, incubated the samples at room temperature for 10 min and centrifuged them at 12,000 ×g for 10 min at 4°C. Finally, the RNA pellet was washed by 1 ml of 75% ethanol, air-dried, suspended in RNase-free water and water-bathed at 60°C for 10 min. The quality of total RNA was evaluated and controlled by Agilent 2100 Bioanalyzer. For each sample we replicated total RNA isolation for three times and pooled them for cDNA library construction and sequencing.

### cDNA library construction and sequencing

A total amount of 20 μg RNA was used for transcriptome cDNA library construction by using TruSeq^TM^ RNA Sample Preparation Kit v2 (Illumina) and the cDNA library was sequenced on an Illumina HiSeq 2000 platform following the manufacturers’ protocols. In brief, poly(A) mRNAs were obtained by using Dynal Oligo(dT) beads (Invitrogen). mRNAs were then chemically fragmented into ~200 nt fragments. mRNA fragments were next copied into first strand cDNA by using reverse transcriptase and random primers, followed by the second strand cDNA synthesis using DNA Polymerase I (Invitrogen) and RNase H (Invitrogen) treatment. After end repaired by using End Repair Mix (Illumina) reagent, the cDNA fragments were purified and enriched to create the final cDNA library. A total of six libraries (each sample has two replicates) were sequenced by pair-end (2×90 bp) method on an Illumina HiSeq^TM^ 2000 platform.

### *De novo* assembly of the transcriptome

After adapter sequences and low quality reads were removed, raw sequencing reads were cleaned and quality controlled by FastQC software (http://www.bioinformatics.babraham.ac.uk/projects/fastqc/). Then, *de novo* assembly of high quality reads was carried out by Trinity software (release 2014-07-17) [37], according to the protocol [38].

### Transcriptome annotation

Gene Ontology (GO) and biological pathway annotations for the assembled transcriptome were performed by mapping them to public databases, including NCBI non-redundant (NR), UniProt and Kyoto Encyclopedia of Genes and Genomes (KEGG) databases. First, BLAST software [39] was used to align all the transcripts to NR and UniProt databases. Matched transcripts were filtered by using a cut-off of e-value (1 × 10^−5^). Then, BLAST2GO software [40] was used to retrieve associated GO items describing biological processes (BPs), cellular components (CCs) and molecular functions (MFs) for the assembled transcripts. The enzyme commission numbers (EC) for each transcript were also annotated by BLAST2GO. Using a cut-off of e-value (≤1e-5), the transcripts with corresponding ECs were obtained and mapped to KEGG metabolic pathway database. Then, likely protein sequences were extracted from the assembled transcripts by TransDecoder, which is included in the Trinity software distribution. Potential signal peptides, transmembrane domains and rRNA transcripts were predicted by using SignalP [41], TMHMM Sever v2.0 [42] and RNAMMER [43], respectively. To identify the proteins distributed in EuKaryotic Orthologous Groups (KOG), Clusters of Orthologous Groups (COGs), and non-supervised orthologous groups (NOGs), the likely protein sequences were further used to search against EggNOG database (v4.1, http://eggnogdb.embl.de) [44]. Protein functional domains were identified by mapping the likely proteins to Pfam database [45] using HMMER [46] and filtered by using the cut-off of e-value (≤1e-5).

### Reads alignment and transcriptome profile

To profile gene expression in loranthus seeds, Bowtie2 [47] and RSEM (RNA-Seq by Expectation-Maximization) [48] were used to map clean reads of CK (CK-1 and CK-2), Tac-16 (Tac-16-1 and Tac-16-2) and Tac-36 (Tac-36-1 and Tac-36-2) to the assembled transcriptome and evaluate the abundance of each transcript, respectively. To compare the expression levels of transcripts in different samples, we used FPKM (fragments per kilobase of transcript per million mapped reads) for normalization and to present the expression of transcripts.

### Differentially expressed transcripts

Transcripts differentially expressed during the dehydration process were identified by using egdeR [49]. We used a strict criterial to select differentially expressed transcripts: normalized expression >1 FPKM, Log2FC (log2 fold change) >1 (up-regulated) or Log2FC <-1 (down-regulated), *p-value* <0.05 and FDR (false discovery rate) <0.05.

### GO and KEGG pathway enrichment analysis

To identify significant GO terms and KEGG pathways enriched by candidate transcripts, we used *p-value* (Fisher’s exact test) to show the significance of enrichment for specific GO term. Then, *q-value* [50] was calculated to correct the *p-value* for each GO term or pathway and control the false discovery rate. Significant GO items and KEGG pathways should satisfy the critical: *q-value*<0.05. GO items and KEGG pathways not associated with plant bio activities were filtered.

### Quantitative real-time PCR

To validate the expression of the assembled transcripts, quantitative real-time PCR (qRT-PCR) experiment was performed following the protocols. In brief, total RNA was extracted by using TRIzol^®^ reagent (Invitrogen), as described. The quality of total RNA was evaluated and controlled by NanoDrop 1000 (Thermo Scientific). Forward and reverse primer sequences for candidate transcripts and control (*actin-3*) were predicted by Primer3 (http://bioinfo.ut.ee/primer3-0.4.0/) and synthesized at BGI-Shenzhen. Then, FastQuant RT Kit (with gDNase, Tiangen) was used to synthesize cDNA from total RNA (1 μg). The cDNA samples (100 ng, 2 μl) were next mixed with 10 μl of SuperReal PreMix (SYBR Green, Tiangen), forward primer (0.8 μl), reverse primer (0.8 μl), 5 × gDNA Buffer (2 μl) and ddH_2_O (6.4 μl) to make the final PCR reaction mix. The final PCR reaction mix (SYBR Green) was amplified on a LightCycler480II (Roche) with three steps PCR, including 1 cycle initial denaturation (95°C for 3 min), 40 cycles of PCR reactions (95°C for 10 s, 60°C for 15 s and 72°C for 20 s) and a melting/dissociation curve stage (95°C for 10 s, 65°C for 1 min and a continuous temperature ramp (0.11°C/s) from 65 to 97°C). For each candidate we replicated three times of qRT-PCR in every sample. Average of the Ct (cycle threshold) for each candidate was calculated, ΔCt was used to evaluate the expression levels of candidate transcripts in each sample and ΔΔCt method was used to show the different expression of a particular transcript between two samples [51].

$$\Delta \Delta C_{t}=(C_{t}^{t1}-C_{t}^{r1})-(C_{t}^{t0}-C_{t}^{r0})$$ (3)

Whereas $C_{t}^{t1}$ and $C_{t}^{t0}$ stand for the average Ct values of a candidate transcript detected in dehydrated seeds and fresh seeds, respectively; $C_{t}^{r1}$ and $C_{t}^{r0}$ stand for the average Ct values of actin-3 detected in dehydrated seeds and fresh seeds, respectively. The relative normalized expression (RNE) was calculated by using 2^-ΔΔCt^ method.

### Availability of data and material

The raw sequencing files of these six samples (FASTQ formatted files) can be accessed in the NCBI Sequence Read Archive (SRA) database (http://trace.ncbi.nlm.nih.gov/Traces/sra/) under the accession number of SRA309567 (CK-1: SRR2902061; CK-2: SRR4067162; Tac-16-1: SRR2902062; Tac-16-2: SRR4067163; Tac-36-1: SRR2902063; Tac-36-2 SRR4067164). The assembled transcripts can be accessed from NCBI Transcriptome Shotgun Assembly Sequence (TSA) Database under the accession number GELW00000000. Scripts for key steps, such as transcriptome de novo assembly, annotation, expression profile and differential expression, can be seen in **S1 File**.

## Results and Discussion

### Seeds dehydration, viability test and germination experiment

**Fig 1:**
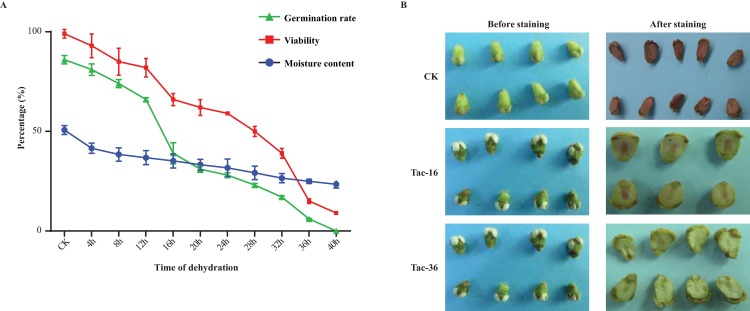
Treatment of loranthus seeds. (**A**) Moisture content assay, viability test and germination test of loranthus seeds. (**B**) TTC test of CK, Tac-16 and Tac-36 show fresh seeds (CK) are viable, seeds in Tac-16 group are abnormal and seeds in Tac-36 group are non-viable.

The loranthus seeds were collected from 10 trees of *Dracontomelon duperreanum* Pierre in Guangxi Province of China in December of 2014 and confirmed by senior botanists. Loranthus is wildly used in traditional Chinese medicine prescriptions and it is still unknown about the loranthus seed response to environmental stresses. Loranthus seeds always disperse as fresh seeds in wild by our long-term observations, so among the environmental stresses we first want to study the gene expression changes in loranthus seeds in response to water loss. Initially, we observed that loranthus seeds were recalcitrant because the viability and germination rate of loranthus seeds dropped quickly if they were stored in dry environment [52]. Using seed germination experiment we found after being stored for three days the germination rate decreased from 86% (germination rate of fresh seeds) to 40% and after six days the germination rate was only 5%. Then, we examined the moisture content of fresh loranthus seeds was 50.7% (w/w) on average (**Fig 1A**), which is similar to that in soybean [53]. Next, we performed the dehydration experiment and tested the viability of loranthus seeds (25 seeds × 4 replicates) using TTC staining. **Fig 1A** showed the moisture content, the viability and germination rate of loranthus seeds (25 seeds × 4 replicates) during the dehydration process. It is clear that the viability and germination rate of loranthus seeds were associated with the water content in seeds. The germination rate of loranthus fresh seeds was 86% (viability: 99%) and after being dehydrated for 16 hours it was dropped to 66% (viability: 66%, moisture content: 35.17%). If the seeds were dehydrated for 36 hours, the moisture content was decreased to 24.93%, the viability was decreased to 15% and the germination rate was 6%. After 40 hours’ dehydration the moisture content, viability and germination rate were examined as 23.47%, 9% and 0, respectively. Because both viability and germination rate were decreased most after being dehydrated for 16 hours (Tac-16) and 36 hours (Tac-36), we collected seeds from these two time points and CK to study the gene expression changes of loranthus seeds during the dehydration process. TTC staining of CK, Tac-16 and Tac-36 confirmed that the viability of loranthus seeds was affected by drought (**Fig 1B**). Loranthus seeds were abnormal after 16 hours’ dehydration and non-viable after 36 hours’ dehydration. In fact, moisture content has been experimented to play a key role in seed germination [54, 55], especially in early seed germination [56]. In *Arabidopsis*, water in seeds supplies several biochemical reactions during the seed germination, such as water up-taken from outside [53, 56]. At molecular level, drought stress induced genes have been characterized in different plant species including *Arabidopsis* [14, 15], rice [16–18], soybean [19, 20], and other plants [21–23].

### Transcriptome *de novo* assembly

**Fig 2:**
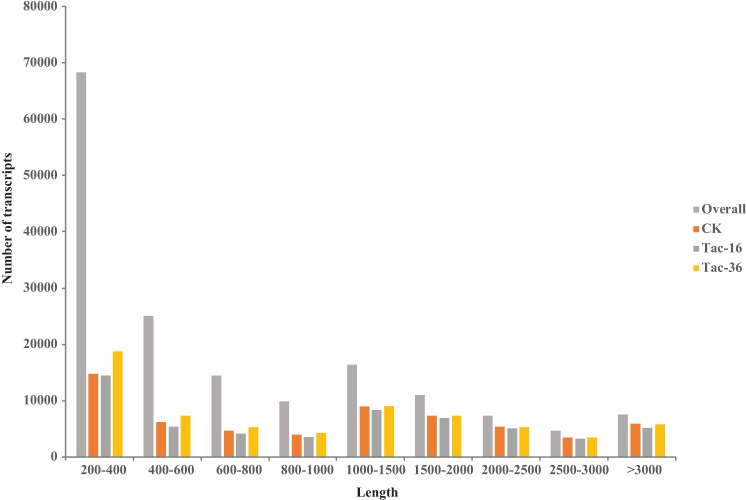
Length distribution of the assembled transcripts and transcripts (> 1FPKM) detected in CK, Tac-16 and Tac-36.

In this study, we conducted six cDNA libraries (in biological replicates) for CK, Tac-16 and Tac-36 and sequenced them using paired-end transcriptome sequencing. After the low quality reads were removed, 386,542,846 high quality reads were obtained and used for *de novo* assembly by using Trinity software [37]. A total of 164,546 transcripts corresponding to 114,971 genes (**Table 1**) were assembled. The assembly contains 149,031,959 bases (159 M in size), which was determined to be ‘strong’ and ‘fully consider’ for evaluating *de novo* transcriptome [57]. Next, other measures like GC content, N10, N20 and N50 were used to evaluate the transcriptome. The GC content, N10, N20 and N50 of the assembled transcriptome were calculated as 41.12%, 3,972, 3,040 and 1,610, respectively (**Table 1**). Of them, N50 is a statistical measure of average length of a set of sequences like genome and transcriptome sequences [58]. In addition, length distribution of the assembled transcripts (**Fig 2**) told us there were a total of 46,869 (28.48%) transcripts longer than 1,000 bp, of them 7,521 (4.57%) transcripts were longer than 3,000 bp. Except short transcripts (< 400 nt), the numbers of assembled transcripts, transcripts detected in CK, Tac-16 and Tac-36 were close to each other. This is the first time to study the loranthus seed transcriptome, it is hard to evaluate the numbers of transcripts and genes in loranthus due to the missing information of its genome sequence and annotation.

**Table 1:** Overview of the transcriptome *de novo* assembly.

Type	Result
High quality reads	386,542,846
Total Trinity genes	114,971
Total Trinity transcripts	164,546
GC (%)	41.12
N10	3,972
N20	3,040
N50	1,610
Total assembled bases	149,031,959

### Annotation of the assembled loranthus seed transcriptome

**Fig 3:**
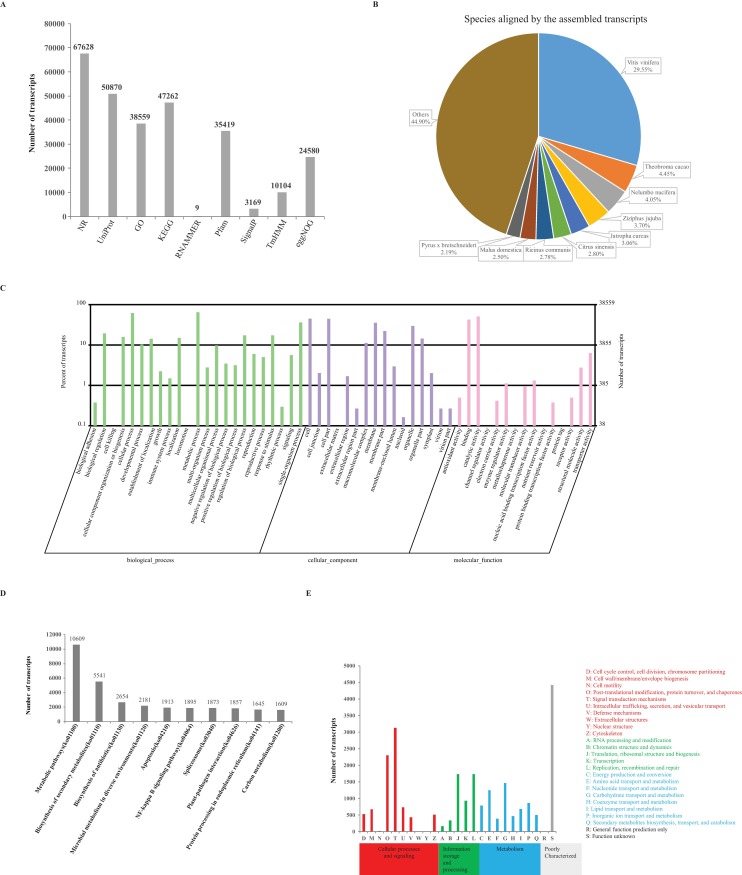
Annotation of the assembled transcriptome. (**A**) Number of transcripts aligned to different databases. (**B**) Species aligned by the assembled loranthus seed transcriptome. (**C**) Gene Ontology analysis for the assembled loranthus seed transcriptome. (**D**) Top 10 significant KEGG pathways. (**E**) COG annotation.

We next annotated the assembled transcriptome by mapping it to NCBI non-redundant (NR), UniProt, GO and KEGG databases and the numbers of transcripts matched to each database can be seen in **Fig 3A**. By using BLAST software [39] and a cut-off of e-value (< 1 × e^-5^), the largest number (67,628, 41.10%) of transcripts were aligned to NR database, followed by the UniProt database (50,870, 30.92%). We further explored the loranthus transcripts aligned to species in the NR mapping results (**Fig 3B)**. There were 19,977 transcripts aligned to *Vitis vinifera*, taking 29.54% of all the transcripts aligned to NR database, followed by *Theobroma cacao* (3,010, 4.45%), *Nelumbo nucifera* (2,742, 7.05%) and *Ziziphus jujuba* (2,504, 3.70%). Using the NR and UniProt mapping results 38,559 (23.43%) transcripts (**Fig 3A**) whose orthologous sequences have GO annotations were divided into three categories: cellular component, biological process, and molecular function (**S1 Table**). GO analysis (**Fig 3C)** showed 19 GO items were enriched by more than 10% of the total assembled transcripts. Top 10 of them were “metabolic process” (25,062 transcripts), “cellular process” (23,847 transcripts), “catalytic activity” (19,620 transcripts), “cell” (17,340 transcripts), “cell part” (17,196 transcripts), “binding” (16,406 transcripts), “single-organism process” (13,988 transcripts), “membrane” (13,668 transcripts), “organelle” (11,457 transcripts) and “membrane part” (8,590 transcripts). In addition, 47,262 transcripts were identified to involve in 362 different KEGG pathways (**Fig 3A**). According to the numbers of transcripts, top 10 KEGG pathways can be seen in **Fig 3D**. The most significant KEGG pathway was “metabolic pathway” (ko01100), containing 10,609 transcripts. We also identified 1,857 and 1,645 transcripts playing a key role in the pathways of “plant-pathogen interaction” (ko04626) and “plant hormone signal transduction” (ko04075), respectively. The ontologies and pathways annotated for loranthus seed transcriptome showed their potential functions in seed development [59] and tolerance of environmental stresses [60]. Interestingly, 9 transcripts (**Fig 3A**) were predicted to be ribosomal RNAs by using RNAMMER [43].

Next, we extracted likely proteins from the assembled transcripts using TransDecoder. In total, 49,004 transcripts (29.78% of the total assembled transcripts) were predicted to encode 61,610 proteins. Among the likely proteins, we identified 40,846 (24.82%) transcripts containing Pfam domain sequences, 3,169 (1.93%) with signal peptides and 10,104 (6.14%) transcripts encoding membrane related proteins (**Fig 3A**). Then, likely proteins encoded by the assembled transcripts were mapped to eggNOG database and proteins encoded by 24,580 (14.94%) transcripts were distributed in EuKaryotic Orthologous Groups (KOG), Clusters of Orthologous Groups (COGs), and non-supervised orthologous groups (NOGs), see **S2 Table**. As show in **Fig 3E**, 4,315 likely proteins were poorly characterized, 2,911 likely proteins were from COG of signal transduction mechanisms, and 2,138 likely proteins were from COG of post-translational modification, protein turnover, and chaperones. Annotations from different perspectives will give a better understanding of the functions of the assembled transcripts and help to identify transcripts involved in the dehydration process. In addition, the reasons of some transcripts annotated without encoding ability should be further explored [61].

### Transcriptome profile and different expression

**Fig 4:**
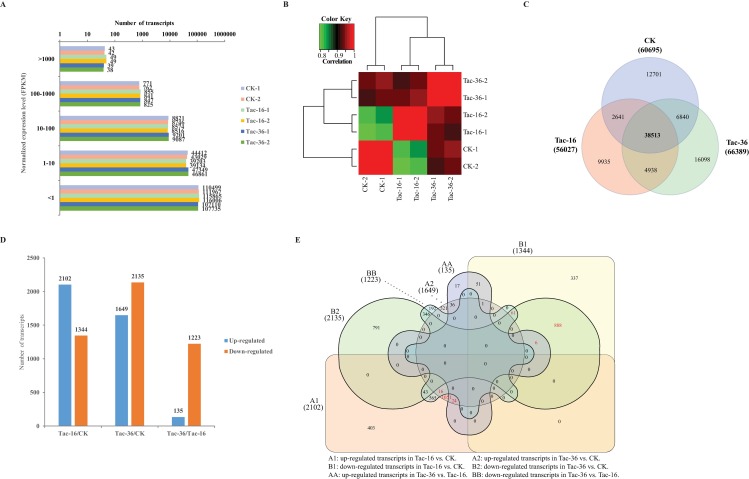
Transcriptome profiling and differential expression. (**A**) Distribution of normalized expression of transcripts detected in all samples. (**B**) Heat map of correlations between replicates. (**C**) Venn diagram of transcripts (>1 FPKM) detected in CK, Tac-16 and Tac-36. (**D**) Number of up- and down-regulated transcripts in Tac-16 and Tac-36 compared to CK. (**E**) Venn diagram of differentially expressed transcripts identified in all comparisons. Numbers in red represent commonly up- (1091) and down-regulated (955) transcripts in Tac16 and Tac-36 compared to CK.

The viability and germination rate of loranthus seeds dropped quickly during dehydration (**Fig 1A**). In order to identify genes induced by drought stress and profile them in loranthus seeds, we performed two biological replicates for CK, Tac-16 and Tac-36 samples and the expression of transcripts was evaluated separately in each replicate. Bowtie2 [47] was used to align the high quality reads to the assembled transcriptome and RSEM [48] tool was used to profile gene expression in all samples. In total, we obtained 91,666 transcripts (>1 FPKM) and 54,047, 52,579, 48,681, 48,540, 57,436 and 56,811 transcripts (>1 FPKM) distributed in CK-1. CK-2, Tac-16-1, Tac-16-2, Tac-36-1 and Tac-36-2, respectively. Length distribution of transcripts detected in CK, Tac-16 and Tac-36 can be found in **Fig 2**and the distribution of their normalized expression (**Fig 4A**) showed 80.53% ~ 82.49% of the total detected transcripts (excluding transcripts < 1 FPKM) in each sample were less than 10 FPKM. Pearson correlations for the replicates were above 0.9 in CK, Tac-16 and Tac-36 well (**Fig 4B**), which indicated the replicates were performed very well. Venn diagram (**Fig 4C**) of detected transcripts in CK, Tac-16 and Tac-36 (> 1 FPKM in at least one of the two replicates) revealed 38,513 (42.01% of the total detected transcripts) were commonly detected and 12,701 (20.93% of transcripts detected in CK), 9,935 (17.73% of transcripts detected in Tac-16) and 16,098 (24.74% of transcripts detected in Tac-36) transcripts were detected exclusively in CK, Tac-16 and Tac-36, respectively.

To characterize drought induced genes in loranthus seeds, we employed edgeR [49] to identify transcripts differentially expressed in Tac-16 and Tac-36 compared to CK and used the criterial as follows: FPKM >5 in at least one sample, Log2FC >1 or Log2FC <-1, *p-value* <0.05 and FDR <0.05. Compared to CK, we obtained 2,102 up-regulated and 1,344 down-regulated transcripts in Tac-16 and 1,649 up-regulated and 2,135 down-regulated transcripts in Tac-36 (**Fig 4D**). We also compared Tac-16 and Tac-36 and found 1,358 transcripts differentially expressed including 135 transcripts up-regulated in Tac-36 and 1,223 transcripts up-regulated in Tac-16 (**Fig 4D**). In all three comparisons we obtained a total of 5,349 transcripts differentially expressed in loranthus seeds during the dehydration. Venn diagram of differentially expressed transcripts (**Fig 4E**) showed the numbers of transcripts commonly and specifically up-regulated or down-regulated in Tac-16 and Tac-36. It is revealed that 1,091 transcripts were commonly up-regulated and 955 transcripts were commonly down-regulated in Tac-16 and Tac-36 in comparison of CK. Interestingly, 24 transcripts such as c36451_g2_i1 (LEGB4_VICFA, legumin type B), c51416_g2_i1 (AB40G_ARATH, ABC transporter G family member 40) and c59053_g2_i1 (DIR23_ARATH, dirigent protein 23) kept increasing while 61 transcripts such as c60985_g1_i1 (12KD_FRAAN, auxin-repressed 12.5 kDa protein), c67927_g8_i3 (BH094_ARATH, transcription factor bHLH94), c49978_g1_i1 (DRE2D_ARATH, dehydration-responsive element-binding protein 2D) and c39272_g1_i1 (HS23C_OXYRB, small heat shock protein, chloroplastic) kept decreasing during the dehydration process. Detailed information of differentially expressed transcripts in Tac-16 and Tac-36 compared to CK can be accessed in **S3 Table**.

### Functional analysis of differentially expressed transcripts

Next, we annotated the differentially expressed transcripts using GO and KEGG pathway databases. Overall, loranthus seed transcripts induced by drought were involved in metabolic pathways, such as “Metabolic pathways” (ko01100), “Riboflavin metabolism” (ko00740) and “Terpenoid backbone biosynthesis” (ko00900), signaling transduction pathway “Plant hormone signal transduction” (ko04075) and environmental adaption pathways such as “Plant-pathogen interaction” (ko04626), “Circadian entrainment” (ko04713) and “Circadian rhythm–plant” (ko04712) (**Table 2**).In addition, we found several pathways may be associated with loranthus seeds in response to dehydration at different stages. For example, there were 107 transcripts involved in “Ribosome” (ko03010) in Tac-16 (*p-value* = 2.2E-16, *q-value* = 6.8E-14) but only 40 in Tac-36 (*p-value* = 0.683, *q-value* = 1). And transcripts involved in “Neuroactive ligand-receptor interaction” (ko04080) decreased from 19 in Tac-16 (*p-value* = 1.37E-15, *q-value* = 4.24E-13) to 0 in Tac-36 (*p-value* = 1, *q-value* = 1). Although the numbers of transcripts annotated by KEGG pathway were decreased in Tac-36 compared to Tac-16, pathways of “Biosynthesis of secondary metabolites” (ko01110), “Biosynthesis of antibiotics” (ko01130) and “plant hormone signal transduction” (ko04075) had more differentially expressed transcripts in Tac-36 (**Table 2**).

**Table 2:** KEGG pathway analysis for differentially expressed transcripts.

Group	Pathway	ID	Tac-16^a^	P-value	Q-value	Tac-36^b^	P-value	Q-value
Global and overview maps	Metabolic pathways	ko01100	426	2.2E-16	6.8E-14	572	2.2E-16	7.11E-14
Global and overview maps	Biosynthesis of secondary metabolites	ko01110	232	1.07E-13	3.3E-11	391	2.2E-16	7.11E-14
Signal transduction	Plant hormone signal transduction	ko04075	87	3.03E-12	9.35E-10	118	2.2E-16	7.11E-14
Carbohydrate metabolism	Galactose metabolism	ko00052	44	4.19E-12	1.29E-09	48	2.16E-12	6.97E-10
Biosynthesis of other secondary metabolites	Flavonoid biosynthesis	ko00941	11	0.005321	1	26	9.56E-12	3.09E-09
Carbohydrate metabolism	Starch and sucrose metabolism	ko00500	68	1.42E-07	4.38E-05	86	2.21E-11	7.14E-09
Biosynthesis of other secondary metabolites	Phenylpropanoid biosynthesis	ko00940	27	0.002195	0.678255	48	1.31E-10	4.23E-08
Carbohydrate metabolism	Amino sugar and nucleotide sugar metabolism	ko00520	60	9E-12	2.78E-09	61	6.16E-10	1.99E-07
Glycan biosynthesis and metabolism	Other glycan degradation	ko00511	27	1.29E-05	0.003974	37	3.12E-09	1.01E-06
Metabolism of other amino acids	Cyanoamino acid metabolism	ko00460	17	0.009255	1	33	4.59E-09	1.48E-06
Global and overview maps	Biosynthesis of antibiotics	ko01130	95	0.001081	0.334029	132	5.25E-09	1.7E-06
Environmental adaptation	Plant-pathogen interaction	ko04626	86	2.19E-07	6.77E-05	99	1.85E-08	5.98E-06
Biosynthesis of other secondary metabolites	Monobactam biosynthesis	ko00261	5	0.03746	1	14	1.92E-08	6.2E-06
Global and overview maps	Microbial metabolism in diverse environments	ko01120	95	7.67E-07	0.000237	111	2.89E-08	9.34E-06
Energy metabolism	Photosynthesis—antenna proteins	ko00196	0	1	1	9	3.46E-08	1.12E-05
Amino acid metabolism	Lysine biosynthesis	ko00300	2	0.5836	1	14	4E-08	1.29E-05
Metabolism of terpenoids and polyketides	Carotenoid biosynthesis	ko00906	9	0.003549	1	17	7.15E-08	2.31E-05
Environmental adaptation	Circadian entrainment	ko04713	16	2.07E-06	0.000639	19	1.18E-07	3.8E-05
Metabolism of cofactors and vitamins	Porphyrin and chlorophyll metabolism	ko00860	6	0.4873	1	23	1.37E-07	4.43E-05
Xenobiotics biodegradation and metabolism	Naphthalene degradation	ko00626	3	0.02358	1	8	2.85E-07	9.19E-05
Xenobiotics biodegradation and metabolism	Polycyclic aromatic hydrocarbon degradation	ko00624	15	1.32E-08	4.09E-06	14	4.91E-07	0.000158
Biosynthesis of other secondary metabolites	Stilbenoid, diarylheptanoid and gingerol biosynthesis	ko00945	14	3.4E-06	0.00105	16	6.4E-07	0.000207
Metabolism of other amino acids	Taurine and hypotaurine metabolism	ko00430	5	0.002268	0.700812	9	7.97E-07	0.000257
Cell growth and death	Apoptosis	ko04210	66	0.01266	1	94	1.55E-06	0.000499
Environmental adaptation	Circadian rhythm—plant	ko04712	20	0.000174	0.053828	26	1.94E-06	0.000627
Biosynthesis of other secondary metabolites	Anthocyanin biosynthesis	ko00942	1	0.5807	1	8	4.24E-06	0.001368
Excretory system	Aldosterone-regulated sodium reabsorption	ko04960	0	1	1	10	4.46E-06	0.001442
Biosynthesis of other secondary metabolites	Isoflavonoid biosynthesis	ko00943	5	0.0185	1	10	8.61E-06	0.002782
Metabolism of cofactors and vitamins	Riboflavin metabolism	ko00740	2	0.4556	1	10	8.61E-06	0.002782
Global and overview maps	Degradation of aromatic compounds	ko01220	5	0.002567	0.793203	8	1.06E-05	0.003424
Lipid metabolism	Glycerolipid metabolism	ko00561	16	0.09111	1	30	1.38E-05	0.00447
Excretory system	Vasopressin-regulated water reabsorption	ko04962	8	0.001223	0.377907	11	2.74E-05	0.008863
Carbohydrate metabolism	Glycolysis / Gluconeogenesis	ko00010	42	2.4E-05	0.00741	46	2.8E-05	0.00905
Digestive system	Mineral absorption	ko04978	5	0.1158	1	12	3.83E-05	0.012368
Lipid metabolism	Fatty acid biosynthesis	ko00061	10	0.04153	1	18	4.29E-05	0.013841
Metabolism of terpenoids and polyketides	Terpenoid backbone biosynthesis	ko00900	15	0.003843	1	21	4.3E-05	0.013886
Xenobiotics biodegradation and metabolism	Dioxin degradation	ko00621	3	0.000889	0.274577	4	4.92E-05	0.015888
Metabolism of terpenoids and polyketides	Limonene and pinene degradation	ko00903	12	2.78E-05	0.008581	12	9.9E-05	0.031971
Global and overview maps	2-Oxocarboxylic acid metabolism	ko01210	10	0.3284	1	23	0.000112	0.036079
Xenobiotics biodegradation and metabolism	Bisphenol degradation	ko00363	12	2.06E-06	0.000637	10	0.000214	0.06909
Carbohydrate metabolism	Citrate cycle (TCA cycle)	ko00020	22	1.75E-05	0.00542	21	0.000326	0.105266
Metabolism of other amino acids	Glutathione metabolism	ko00480	21	3.74E-06	0.001157	17	0.001681	0.542963
Carbohydrate metabolism	Ascorbate and aldarate metabolism	ko00053	18	1.84E-05	0.00567	15	0.002348	0.758404
Xenobiotics biodegradation and metabolism	Aminobenzoate degradation	ko00627	12	5.62E-05	0.017366	10	0.002609	0.842707
Translation	Ribosome	ko03010	107	2.2E-16	6.8E-14	40	0.683	1
Digestive system	Protein digestion and absorption	ko04974	25	2.2E-16	6.8E-14	4	0.3388	1
Signaling molecules and interaction	Neuroactive ligand-receptor interaction	ko04080	19	1.37E-15	4.24E-13	0	1	1
Sensory system	Phototransduction—fly	ko04745	20	3.63E-11	1.12E-08	9	0.009196	1
Sensory system	Phototransduction	ko04744	12	5.86E-08	1.81E-05	6	0.01012	1

a. Number of differentially expressed transcripts in Tac-16 compared to CK.

b. Number of differentially expressed transcripts in Tac-36 compared to CK.

**Fig 5:**
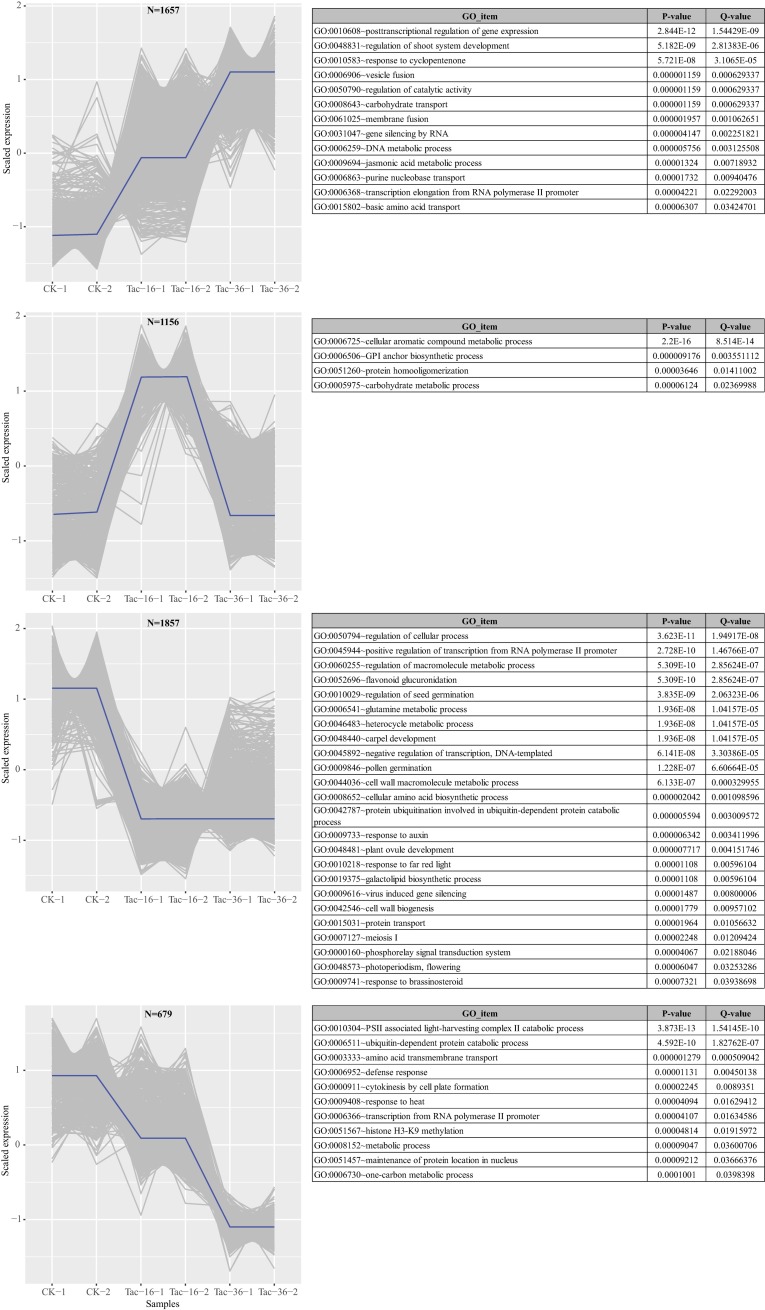
K-means clustering analysis of the differentially expressed transcripts in dehydrated loranthus seeds in comparison of CK and GO analysis.

We performed K-means clustering analysis using MEV software (v4.9) and found four main groups of transcripts with different expression patterns in CK, Tac-16 and Tac-36 (**Fig 5B**). The biggest group containing 1,857 transcripts that were down-regulated in loranthus dehydrated seeds compared to CK. They were significantly enriched (*p-value* <0.05, *q-value* <0.05) in biological processes including “regulation of cellular process” (GO:0050794), “regulation of seed germination” (GO:0010029), “pollen germination” (GO:0009846), “response to auxin” (GO:0009733), “plant ovule development” (GO:0048481), “response to far red light” (GO:0010218), “photoperiodism, flowering” (GO:0048573) and “response to brassinosteroid” (GO:0009741). The decreased expression of transcripts involved in seed germination and cell development, especially the plant ovule development indicated the germination of loranthus seeds was affected due to the water loss, so we assume that loranthus seeds are recalcitrant. The second group contained 1657 transcripts that were up-regulated in loranthus seeds during the dehydration. GO analysis revealed these transcripts were involved in the biological processes such as “posttranscriptional regulation of gene expression” (GO:0010608), “regulation of shoot system development” (GO:0048831), “response to cyclopentenone” (GO:0010583), “vesicle fusion” (GO:0006906), “regulation of catalytic activity” (GO:0050790) and “jasmonic acid metabolic process” (GO:0009694). The third group contained 1,156 transcripts whose expression peaked in Tac-16. They were involved in “cellular aromatic compound metabolic process” (GO:0006725), “GPI anchor biosynthetic process” (GO:0006506), “protein homooligomerization” (GO:0051260) and “carbohydrate metabolic process” (GO:0005975). The last group contained 679 transcripts that were down-regulated in loranthus seeds during the dehydration process. Compared to the biggest group, the transcripts in this group were down-regulated in Tac-36 compared to Tac-16 and were involved in the biological processes like “defense response” (GO:0006952), “cytokinesis by cell plate formation” (GO:0000911), “response to heat” (GO:0009408), “transcription from RNA polymerase II promoter” (GO:0006366) and “metabolic process” (GO:0008152). Overall, in response to drought loranthus seeds actively or passively reduced the cell developmental activities, metabolism and transcripts involved in seed germination and plant defense system while transcripts associated with posttranscriptional regulation and gene silencing by RNA were up-regulated due to the water loss in loranthus seeds. In tobacco, reducing the metabolism is expected to play a major role at the early stage in programmed cell death [62]. Auxin and other hormones like abscisic acid (ABA), cytokinins and ethylene are well known to regulate the cell growth in multiple stages including cell development and cell death [63]. It is interesting that ABA also has the capacity of delaying the process of programmed cell death in aleurone cells although the mechanism is unknown [64, 65]. Another review says cell death is programmed when the metabolism is perturbed by abiotic stresses and the cells seem to be ready to die based on the integration of different signals including auxin, cytokinins, ethylene, and elicitors [66].

### Differentially expressed transcripts in response to drought stress

**Fig 6:**
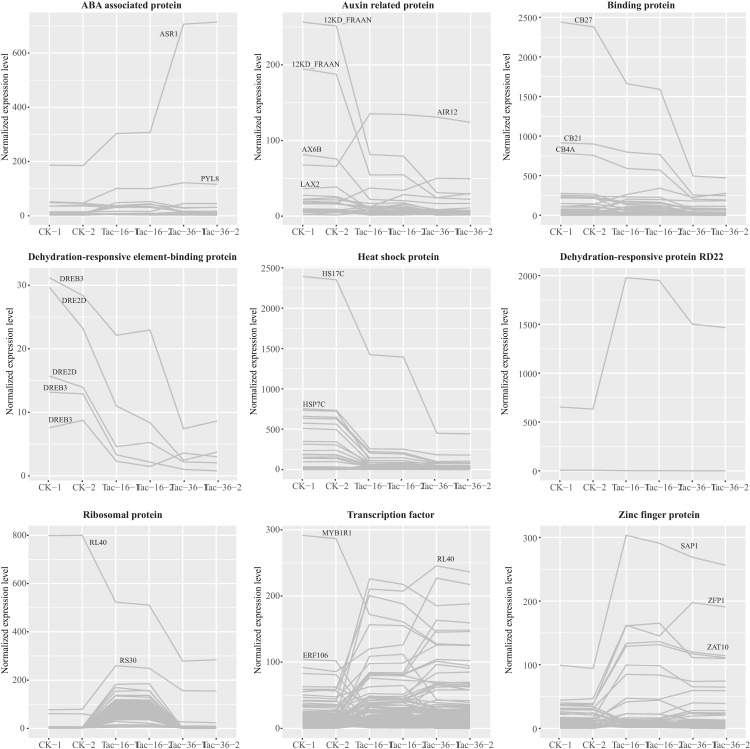
Differentially expressed transcripts involved in response to drought stress.

In order to understand the genes induced by drought in loranthus seeds, we further annotated the differentially expressed transcripts and found there were several genes whose products have been reported to be involved in cell development and cell death. It is well known that ABA-dependent and ABA-independent pathways are two main pathways for plants to respond the water loss [24, 67, 68], so we first examined the expression of ABA associated transcripts. In loranthus seed transcriptome we detected 49 ABA associated transcripts, of which 14 were differentially expressed in dehydrated seeds (**Table 3, Fig 6, S3 Table**). It is notable that ASR1 (Abscisic stress-ripening protein 1), which is induced by water and salt stress [69], was highly expressed (>200 FPKM) and up-regulated significantly in dehydrated loranthus seeds, compared to fresh loranthus seeds.

**Table 3:** Transcripts encoding different proteins are associated with dehydration tolerance.

Gene family	Detected	Differentially expressed	Up-regulated (Tac-16/CK)	Down-regulated (Tac-16/CK)	Up-regulated (Tac-36/CK)	Down-regulated (Tac-36/CK)
ABA associated protein	49	14	6	1	8	4
Auxin related protein	187	27	6	10	5	17
Binding protein	1544	94	37	28	22	34
Dehydration-responsive element-binding protein	12	5	0	3	0	5
Dehydration-responsive protein RD22	11	2	1	0	1	1
Heat shock protein	163	48	18	28	0	25
Late embryogenesis abundant protein	5	0	0	0	0	0
Ribosomal proteins	1049	88	86	0	0	22
Transcriptional factors	1277	160	80	20	75	63
Zinc finger proteins	654	52	25	11	30	19

The functions of drought induced genes include protecting cells and regulating genes for signal transduction [70, 71]. According to their functions, these genes are classified into two groups. The first group of genes encoding proteins probably functions in stress tolerance, like LEA proteins, mRNA-binding proteins and aquaporins. In this study, we identified 5 and 55 transcripts encoding LEA proteins and aquaporins, respectively, but only three transcripts encoding aquaporins were differentially expressed significantly in dehydrated loranthus seeds. However, we found a number of binding proteins differentially expressed (**Fig 6**) and most of them were ATP-associated and RNA binding proteins (**Table 3**, **S3 Table**). The second group of gene products may be involved in further regulation of signal transduction and gene expression, like various transcription factors. In total, we detected 1,277 transcripts encoding transcription factors in all samples, of which 160 were differentially expressed during dehydration process. As shown in **Table 3**, 80 up-regulated and 20 down-regulated transcripts encoding TFs were identified in Tac-16 while 75 up-regulated and 63 down-regulated transcripts encoding TFs were identified in Tac-36, compared to CK. Differentially expressed transcripts encoding transcription factors include MYB, WRKY, and some ethylene-responsive transcription factors (**S3 Table**). These transcription factors have been reported to regulate ABA-dependent and ABA-independent pathways in plants to respond the dehydration stress and other stresses [19, 24, 72–75].

Among the ABA-dependent genes, RD22 (dehydration-responsive protein 22) is induced by drought stress because its promoter region contains a *cis*-acting element and can be recognized by MYB and MYC transcription factors [76]. We found RD22 mRNA was significantly up-regulated in dehydrated seeds compared to fresh seeds (**Fig 6**). Another group of ABA-dependent genes was heat shock protein (HSP). In response to dehydration, smHSPs (small HSPs) and HSPs are up-regulated in flesh fly, *Sarcophaga crassipalpis* [77, 78], the collembolan *Folsomia candida* [79], the eutardigrade *Richtersius coronifer* [80] and *Belgica antarctica* [81]. In contrast, we found smHSPs such as HS22C and HS23C and HSPs such as HS17C and HSP7C were down-regulated under drought stress in loranthus seeds (**Table 3**, **Fig 6**). It is hard to determine the expression patterns of HSPs under drought stress in plants. In *Arabidopsis* five transcripts encoding HSPs were up-regulated while one was down-regulated during rehydration process after dehydration [15]. In current study, we found 18 up-regulated and 28 down-regulated HSP transcripts in Tac-16 compared to CK, but they were down-regulated in loranthus seeds after being dehydrated over 36 hours. In addition, compared to the down-regulated HSPs, the expression levels of up-regulated HSPs in Tac-16 were much lower (**Fig 6**). Considering this we assume that the up-regulation of HSPs in orthodox seeds maybe one effective way to increase the tolerance to drought stress.

We also showed in **Table 3**and **Fig 6**five down-regulated transcripts encoding dehydration responsive element binding proteins (DRE2D and DREB3), which are ABA-independent genes. In *Arabidopsis*, *Zea mays* and *Oryza sativa*, DREB genes have been demonstrated to improve tolerance to drought, salt, cold and heat [82–84]. It is revealed in wheat and barley overexpression of DREB3 can elevate the expression of various stress responsive genes including CBF/DREB and LEA/COR/DHN genes, which means the DREB3 is inducible under drought stress and may help to increase the tolerance of water deficit [85]. However, both DRE2D and DREB3 were down-regulated in loranthus seeds during the dehydration process. In **Table 3**and **Fig 6**, we showed some other differentially expressed transcripts whose products might be associated with drought tolerance, including auxin related proteins, ribosomal proteins (RP) and zinc finger proteins (ZFNs). Of the 187 detected auxin related transcripts four (12KD_FRAAN, AX6B and LAX2) were down-regulated significantly in Tac-36 compared to CK. Although the relationship between 12KD_FRAAN (auxin-repressed 12.5 kDa protein) and drought is still unknown, 12KD_FRAAN has been reported to be correlated with fruit growth [86]. The decrease of auxin related proteins might explain the reduce of cell activities and viability of seeds during dehydration. In rice, overexpression of ZFNs can enhance drought and salt tolerance [87, 88]. But studies of ZFN gene expression changes in response of dehydration stresses are controversial [81, 89]. It is also hard to tell if ZFNs were up- or down-regulated in Tac-16 compared to CK (**Table 3**, **Fig 6**). We identified 25 up-regulated and 11 down-regulated ZFN transcripts in Tac-16, 30 up-regulated and 19 down-regulated ZFN transcripts in Tac-36, and it is implicated that ZFNs might function in drought tolerance at an early stage of dehydration in loranthus seeds. It is notable that the expression of 86 transcripts encoding ribosomal proteins was up-regulated in Tac-16 but down-regulated in Tac-36. Like ZFNs, ribosomal proteins function in early response of dehydration in *Arabidopsis* [90–92].

### Transcripts involved in seed germination

**Fig 7:**
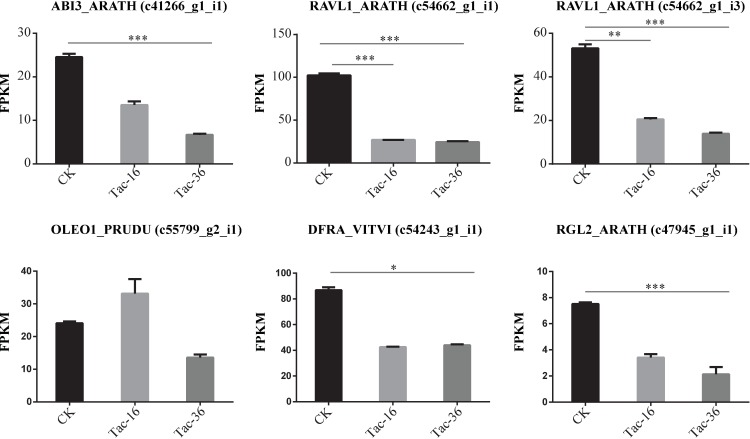
Transcripts associated with seed germination. Different symbols are used show the significance of different expression: < 0.05 (*), <0.01 (**) and <0.001 (***). Error bar represents the standard deviation.

Previous studies have shown several genes are involved in the regulation of seed germination in *Arabidopsis* [93–96], *Brassica oleracea* [97] and *Brassica napus* [98]. Interestingly, some of them have been identified to respond to the drought stress as well. For example, ABI3, controls embryo degreening through SGR1 (Stay-green 1) and SGR2 (Stay-green 2) and participates in ABA-regulated gene expression during seed germination [96, 99]. In addition, COR47 (Cold-induced COR47 protein), OLEO1 (Oleosin), CHI (chalcone flavonone isomerase), CHS (chalcone synthase), DFR (dihydraflavonol-4-reductase), and RAB18 (Ras-related protein 18) have been characterized to regulate the process of *Arabidopsis* seed germination [96]. In rapeseeds, ZFN mRNAs were down regulated during the seed germination. In soybean seeds, LEA mRNAs are inducible by maturation or drying [100]. Some of them have been discussed according to their functions in response to drought stress previously. After removing lowly expressed transcripts strictly (<10 FRPKM), we found the transcripts encoding ABI3_ARATH, RAVL1_ARATH, DFRA_VITVI and RGL2_ARATH were down-regulated significantly in dehydrated loranthus seeds compared to fresh seeds (**Fig 7**). Because of water loss in loranthus seeds, four transcripts encoding CHS2_RUTGR, DFRA_VITVI and RAVL1_ARATH (encoded by two transcripts) were down-regulated while two transcripts encoding OLEO1_PRUDU and RAV2_ARATH increased more or less in Tac-16 and decreased quickly in Tac-36 (**S3 Table**). The down-regulation of seed germination key molecules might be related with the reduced seed viability and low rate of seed germination.

### Validation by quantitative real-time PCR

**Fig 8:**
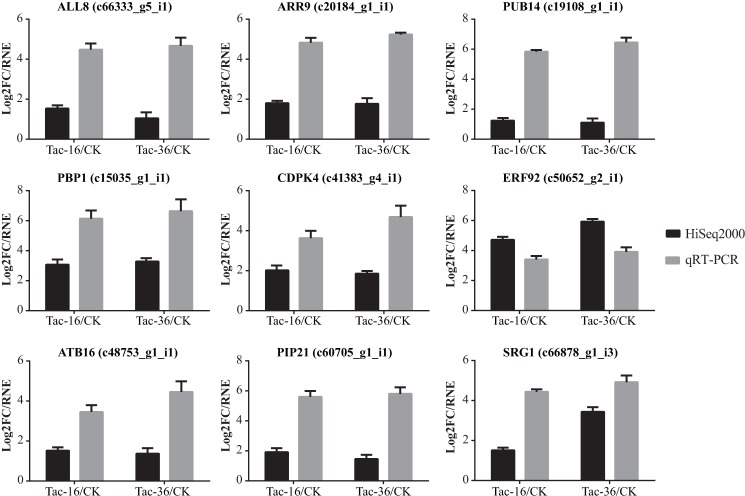
qRT-PCR validation for 9 candidate transcripts. Log2FC means log2 fold change in RNA-Seq experiment while RNE means relative normalized expression (2^-ΔΔCt^) in qRT-PCR experiment. Error bar represents the standard deviation.

To validate the expression of differentially expressed transcripts in dehydrated and fresh loranthus seeds, quantitative real-time PCR (qRT-PCR) experiment was performed due to its high throughput, sensitivity and accuracy. It is widely used to determine the accuracy of transcripts and their expression identified by RNA-Seq [101, 102]. In view of this, 9 transcripts were randomly selected for qRT-PCR and actin-3 was used as control. The expected size of target transcripts ranged from 96 to 200 bp (**S4 Table**). For each transcript we performed three times in every sample. After qRT-PCR amplification ΔCt was calculated. Then, to compare a transcript in different samples, we used ΔΔCt method [51], shown as RNE. **Fig 8**showed the Log_2_FC identified by RNA-Seq (HiSeq2500) and RNE detected by qRT-PCR. It is clear that qRT-PCR confirmed the up-regulation of these transcripts.

## Conclusions

In conclusion, we assembled the transcriptome of seeds during dehydration process in *Taxillus chinensis (DC*.*) Danser* using Illumina RNA-Seq system. A total of 164,546 transcripts corresponding to 114,971 genes were assembled from three libraries–fresh seeds (CK), seeds after 16 hours (Tac-16) and 36 hours (Tac-36) dehydration. Gene expression profiles showed 38,513 transcripts were commonly detected in all samples and 12,701, 9,935 and 16,098 transcripts were detected exclusively in CK, Tac-16 and Tac-36, respectively. Compared to CK, differential expression analysis demonstrated by edgeR characterized 2,102 up-regulated and 1,344 down-regulated transcripts in Tac-16 and 1,649 up-regulated and 2,135 down-regulated transcripts in Tac-36. K-means clustering analysis divided them into four groups with different expression patters and functions. Annotation of differentially expressed transcripts revealed transcripts encoding ABA associated proteins (ASR1 and ABAH4), dehydration-responsive protein RD22, zinc finger proteins and some TFs were up-regulated in Tac-16 and Tac-36. We also found controversial dysregulations of heat shock proteins in response to drought stress in loranthus seeds. Interestingly, transcripts encoding ribosomal proteins peaked in Tac-16, indicating they might be functional in early dehydration process. This is the first time to report loranthus transcriptome. The output of this study will contribute understanding the mechanism of gene regulation in loranthus seeds during dehydration and give new insights of genes induced by drought stress.

## Supporting Information

S1 File Scripts for key steps used in this study. (PDF)

S1 Table Gene Ontology annotation for the assembled transcripts. (XLSX)

S2 Table COG annotation for the assembled transcripts. (XLSX)

S3 Table Differentially expressed transcripts in Tac-36 and Tac-16 compared to CK. (XLSX)

S4 Table Primer sequences used in qRT-PCR experiment. (XLSX)
