# Supplementary material for: Transcriptome Analysis of Taxillusi chinensis (DC.) Danser Seeds in Response to Water Loss
Source: PLoS One. 2017 Jan 3;12(1):e0169177. doi: 10.1371/journal.pone.0169177 (PMC5207531; doi:10.1371/journal.pone.0169177)
Supplement: S1 File — (PDF) [file pone.0169177.s001.pdf]

Supplementary file

Assembl:

Trinity --seqType fq --JM 100G --CPU 8 --left fastq_files/CK_l1_1.fq,fastq_files/Tac-16_l1_1.fq,fastq_files/Tac-36_l1_1.fq, fastq_files/CK2 _1.fq.gz, fastq_files/Tac162 _1.fq.gz, fastq_files/Tac362 _1.fq.gz --right fastq_files/CK_l1_2.fq,fastq_files/Tac-16_l1_2.fq,fastq_files/Tac-36_l1_2.fq, fastq_files/CK2 _2.fq.gz, fastq_files/ Tac162_2.fq.gz, fastq_files/ Tac362_2.fq.gz --output taxillus_chinensis

Annotation:

TransDecoder -t Trinity.fasta

blastx -query Trinity.fasta –db uniprot_sprot.trinotate_v2.0.pep -num_threads 8 -max_target_seqs 1 -outfmt 6 > blastx.outfmt6

blastp -query Trinity.fasta.transdecoder.pep -db uniprot_sprot.trinotate_v2.0.pep -num_threads 8 -max_target_seqs 1 -outfmt 6 > blastp.outfmt6

hmmscan --cpu 8 --domtblout TrinotatePFAM.out Pfam-A.hmm Trinity.fasta.transdecoder.pep >pfam.log

signalp -f short -n signalp.out Trinity.fasta.transdecoder.pep

tmhmm --short < Trinity.fasta.transdecoder.pep >tmhmm.out

perl ~/Trinotate-2.0.2/util/rnammer_support/RnammerTranscriptome.pl --transcriptome Trinity.fasta --transcriptome /usr/local/rnammer/1.2/bin/rnammer

$TRINITY_HOME/util/support_scripts/get_Trinity_gene_to_trans_map.pl Trinity.fasta > Trinity.fasta.gene_trans_map

Trinotate Trinotate.sqlite init --gene_trans_map Trinity.fasta.gene_trans_map --transcript_fasta Trinity.fasta --transdecoder_pep transdecoder.pep

Trinotate Trinotate.sqlite LOAD_swissprot_blastp blastp.outfmt6

Trinotate Trinotate.sqlite LOAD_swissprot_blastx blastx.outfmt6

Trinotate Trinotate.sqlite LOAD_pfam TrinotatePFAM.out

Trinotate Trinotate.sqlite LOAD_tmhmm tmhmm.out

Trinotate Trinotate.sqlite LOAD_signalp signalp.out

Trinotate Trinotate.sqlite LOAD_rnammer Trinity.fasta.rnammer.gff

Trinotate Trinotate.sqlite report -E 1e-5 --pfam_cutoff DNC|DGC|DTC|SNC|SGC|STC > trinotate_annotation_report.xls

RSEM:

perl /usr/local/trinityrnaseq/r20140717-gcc/util/align_and_estimate_abundance.pl --transcripts taxillus_chinensis/Trinity.fasta --est_method RSEM --aln_method bowtie2 --trinity_mode --prep_reference

perl /usr/local/trinityrnaseq/r20140717-gcc/util/align_and_estimate_abundance.pl --transcripts taxillus_chinensis/Trinity.fasta --seqType fq --left fastq_files/CK_l1_1.fq.gz --right

fastq_files/CK_l1_2.fq.gz --est_method RSEM --aln_method bowtie2 --trinity_mode --output_dir RSEM/CK-1

perl /usr/local/trinityrnaseq/r20140717-gcc/util/align_and_estimate_abundance.pl --transcripts taxillus_chinensis/Trinity.fasta --seqType fq --left fastq_files/CK2 _1.fq.gz --right fastq_files/CK2_2.fq.gz --est_method RSEM --aln_method bowtie2 --trinity_mode --output_dir RSEM/CK-2

perl /usr/local/trinityrnaseq/r20140717-gcc/util/align_and_estimate_abundance.pl --transcripts taxillus_chinensis/Trinity.fasta --seqType fq --left fastq_files/Tac-16_l1_1.fq.gz --right fastq_files/Tac-16_l1_2.fq.gz --est_method RSEM --aln_method bowtie2 --trinity_mode --output_dir RSEM/Tac-16-1

perl /usr/local/trinityrnaseq/r20140717-gcc/util/align_and_estimate_abundance.pl --transcripts taxillus_chinensis/Trinity.fasta --seqType fq --left fastq_files/Tac-162_1.fq.gz --right fastq_files/Tac-162_2.fq.gz --est_method RSEM --aln_method bowtie2 --trinity_mode --output_dir RSEM/Tac-16-2

perl /usr/local/trinityrnaseq/r20140717-gcc/util/align_and_estimate_abundance.pl --transcripts taxillus_chinensis/Trinity.fasta --seqType fq --left fastq_files/Tac-36_l1_1.fq.gz --right fastq_files/Tac-36_l1_2.fq.gz --est_method RSEM --aln_method bowtie2 --trinity_mode --output_dir RSEM/Tac-36-1

perl /usr/local/trinityrnaseq/r20140717-gcc/util/align_and_estimate_abundance.pl --transcripts taxillus_chinensis/Trinity.fasta --seqType fq --left fastq_files/Tac-362_1.fq.gz --right fastq_files/Tac-362_2.fq.gz --est_method RSEM --aln_method bowtie2 --trinity_mode --output_dir RSEM/Tac-36-2

Differential expression:

Take Tac-16_vs_CK as an example:

library(edgeR)

x <- read.delim("taxillus_seeds_dehydration.counts.matrix",row.names=" transcript_id")

group <- factor(c(1,1,2,2))

y <- DGEList(counts=x,group=group)

keep <- rowSums(cpm(y)>1) >= 1

y <- y[keep, , keep.lib.sizes=FALSE]

y <- calcNormFactors(y)

et <- exactTest(y,0.1)

temp <- topTags(et,n=Inf)

write.table(temp,"raw_isoform_result.txt")
